# Estimating time-to-onset of adverse drug reactions from spontaneous reporting databases

**DOI:** 10.1186/1471-2288-14-17

**Published:** 2014-02-03

**Authors:** Fanny Leroy, Jean-Yves Dauxois, Hélène Théophile, Françoise Haramburu, Pascale Tubert-Bitter

**Affiliations:** 1 Inserm, CESP Centre for research in Epidemiology and Population Health, U1018, Biostatistics Team, F-94807 Villejuif, France; 2 Univ Paris-Sud, UMRS1018, F-94807 Villejuif, France; 3 Université de Toulouse-INSA, IMT UMR CNRS 5219, Toulouse, France; 4 Département de pharmacologie, Centre de pharmacovigilance, CHU de Bordeaux, Bordeaux, France; 5 INSERM U657, Bordeaux, France

**Keywords:** Pharmacovigilance, Reporting databases, Right truncation, Parametric estimation, Maximum likelihood estimation, Bias, Simulation study

## Abstract

**Background:**

Analyzing time-to-onset of adverse drug reactions from treatment exposure contributes to meeting pharmacovigilance objectives, *i.e.* identification and prevention. Post-marketing data are available from reporting systems. Times-to-onset from such databases are right-truncated because some patients who were exposed to the drug and who will eventually develop the adverse drug reaction may do it after the time of analysis and thus are not included in the data. Acknowledgment of the developments adapted to right-truncated data is not widespread and these methods have never been used in pharmacovigilance. We assess the use of appropriate methods as well as the consequences of not taking right truncation into account (naive approach) on parametric maximum likelihood estimation of time-to-onset distribution.

**Methods:**

Both approaches, naive or taking right truncation into account, were compared with a simulation study. We used twelve scenarios for the exponential distribution and twenty-four for the Weibull and log-logistic distributions. These scenarios are defined by a set of parameters: the parameters of the time-to-onset distribution, the probability of this distribution falling within an observable values interval and the sample size. An application to reported lymphoma after anti TNF- *α* treatment from the French pharmacovigilance is presented.

**Results:**

The simulation study shows that the bias and the mean squared error might in some instances be unacceptably large when right truncation is not considered while the truncation-based estimator shows always better and often satisfactory performances and the gap may be large. For the real dataset, the estimated expected time-to-onset leads to a minimum difference of 58 weeks between both approaches, which is not negligible. This difference is obtained for the Weibull model, under which the estimated probability of this distribution falling within an observable values interval is not far from 1.

**Conclusions:**

It is necessary to take right truncation into account for estimating time-to-onset of adverse drug reactions from spontaneous reporting databases.

## Background

Identifying and preventing adverse drug reactions are major objectives of pharmacovigilance. Owing to design constraints, pre-marketing clinical trials fail to identify rare events, which lead in the last decades to an increased focus placed on the development of post-marketing surveillance methods [1-11]. Post-marketing spontaneous reporting of suspected adverse drug reactions has proved a valuable resource for signal detection [12-17]. It has recently been suggested that the modeling of the time-to-onset of adverse drug reactions could be a useful adjunct to signal detection methods, either from spontaneous reports [18,19] or longitudinal observational data [20]. Timely acquiring knowledge with respect to the time-to-onset distribution of adverse drug reactions contributes to meeting pharmacovigilance objectives. Early estimation procedures tailored to available pharmacovigilance data, *i.e.* spontaneous reporting data, should be sought.

**Figure 1:**
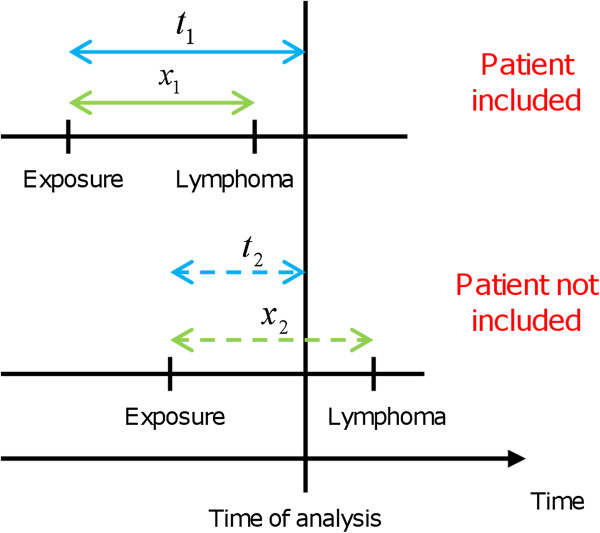
**Right truncation and data on time-to-onset of adverse drug reactions from spontaneous reporting databases.** Some patients who were exposed to the drug and who will eventually develop the adverse drug reaction may do it after the time of analysis. Here, in these hypothetical examples, the patient on the top line is included in the database because he experienced the adverse drug reaction before the time of analysis, *i.e.x*_1 _≤ *t*_1_. The patient on the bottom line is not included in the database because he has not yet experienced the adverse drug reaction, *i.e. *$x_{2}\geq t_{2}$, when data are analyzed.

The data consisting of the time-to-onset among patients who were reported to have potentially developed an adverse drug reaction are right-truncated. Truncation arises because some patients who were exposed to the drug and who will eventually develop the adverse drug reaction may do it after the time of analysis (Figure 1). Among patients exposed to the drug, only those who experienced adverse reactions before time of analysis are included in the database. No information is available for the other patients. If all the patients begin their treatment at the same time, the data are right-truncated with a single truncation time. If they do not all begin their treatment at the same time, the data are right-truncated with different truncation times. In spontaneous reporting, data are right-truncated with different truncation times and they require appropriate statistical methods.

This paper investigates parametric maximum likelihood estimation of the time-to-onset distribution of adverse drug reactions from spontaneous reporting data for different types of hazard functions likely to be encountered in pharmacovigilance. Acknowledgment of the developments adapted to right-truncated data is not widespread and these methods have never been used in pharmacovigilance. No simulation studies are available on the accuracy of their estimates. Furthermore, a naive approach that does not take into account right truncation features of spontaneous reports and uses classical parametric methods instead of appropriate methods may lead to misleading estimates. We consider the two approaches, *i.e.* taking or not taking right truncation into account, and the corresponding parametric maximum likelihood estimators. Both approaches are compared with a simulation study conducted to evaluate the consequences, notably in terms of bias, of not considering right truncation on the maximum likelihood estimates, as well as assessing the performances of the right truncation-based estimation. We also apply these methods to a set of 64 cases of lymphoma occurring after anti TNF- *α* treatment from the French pharmacovigilance.

## Methods

### Proper estimation of the time-to-onset distribution

We consider a given time of analysis and the population of exposed patients who will eventually experience the adverse drug reaction before they die. Let *X* be the time-to-onset of the adverse drug reaction of interest in that population and *F* its cumulative distribution function one is willing to estimate. Observations arising from *n* reported cases are (*x*_1_,*t*_1_),(*x*_2_,*t*_2_),…,(*x*_*n*_,*t*_*n*_), where *x*_*i *_is the time-to-onset calculated as the lag between the time of the occurrence of the reaction and the time of initiation of treatment, and *t*_*i *_is the truncation time calculated as the lag between the time of analysis and the time of initiation of treatment. Let *t*^∗^ be the maximum of the observed truncation times. All observed data meet the condition *x*_*i *_≤ *t*_*i*_.

We consider a parametric model for the time-to-onset *X*, with cumulative distribution function *F *(*x*; *θ*) and density *f*(*x*; *θ*), and derive the following maximum likelihood estimations of *θ*.

When right truncation, *i.e.* the condition *x*_*i *_≤ *t*_*i*_, is ignored, the likelihood of the sample is written as:

$$L_{1}(x_{1},x_{2},\ldots ,x_{n};\theta )=\overset{n}{\underset{i=1}{\prod }}f(x_{i};\theta )\;;$$

maximizing this likelihood yields the naive estimator of *θ*.

When right truncation is considered, the likelihood is modified. Observed times-to-onset consist of *n* independent realizations of random variables with respective distribution the conditional distribution of *X*_*i *_given {*X*_*i *_≤ *t*_*i*_}, that is with cumulative distribution function $\frac{F(x_{i};\theta )}{F(t_{i};\theta )}$ and density $\frac{f(x_{i};\theta )}{F(t_{i};\theta )}$. The likelihood is now written as:

$$L_{2}(x_{1},x_{2},\ldots ,x_{n},t_{1},t_{2},\ldots ,t_{n};\theta )=\overset{n}{\underset{i=1}{\prod }}\frac{f(x_{i};\theta )}{F(t_{i};\theta )}\;;$$

the maximum likelihood estimator from this likelihood, ${\hat{\theta }}_{\text{TBE}}$, is the proper estimation of *θ* and is called the truncation-based estimator (TBE).

The non-parametric maximum likelihood estimation for right-truncated data was developed and used to estimate the incubation period distribution for AIDS [21,22]. However, in a non-parametric setting, one can only estimate the distribution function conditional on the time to event as being less than *t*^∗^ :

$$\hat{\frac{F(x)}{F(t^{*})}}=\underset{v_{j}>x}{\prod }\left(1-\frac{n_{j}}{N_{j}}\right)\;,$$

where the *v*_*j*_’s are the *m* distinct values of the *x*_*i*_’s, *i *=1,…,*n*, taken by $n_{j}=\overset{n}{\underset{i=1}{\sum }}I(X_{i}=v_{j})$ patients and $N_{j}=\overset{n}{\underset{i=1}{\sum }}I(X_{i}\leq v_{j}\leq t_{i})$ for 1 ≤ *j *≤ *m*, *I* denoting the indicator function. The unconditional distribution function is not identifiable, as *F *(*t*^∗^) is not known and cannot be estimated from the data.

In a parametric framework, the unconditional distribution is completely specified by a parameter *θ* of finite dimension. Maximum likelihood estimation of the parameter of interest can be conducted with the conditional distributions that describe the observations and the unconditional distribution can be estimated secondarily by $F(x;{\hat{\theta }}_{\text{TBE}})$. Hence parametric maximum likelihood estimation is potentially more useful than non-parametric estimation since the unconditional distribution is of interest for pharmacovigilance purposes [18,20].

### Simulation study

Some adverse reactions have a very short time-to-onset, from several minutes to several hours after the beginning of treatment. Others occur only after several days, weeks, months or even years of exposure. This variation depends on numerous factors such as the pharmacokinetics of the drug and its metabolites, or the pathophysiological mechanism of the effect. The multiplicity of the underlying mechanisms results in a range of possible hazard functions that can be observed in pharmacovigilance [23]. The simplest model is given by a constant hazard function of time; the corresponding distribution is the exponential distribution with a rate parameter *λ*. Effects may also have an early or a late onset, the latter being the case for instance, when the rate of occurrence of the adverse reaction depends on the duration of exposure. Two distribution families among others make it possible to handle a wide range of hazard functions: the Weibull distributions and the log-logistic distributions (Table 1). Both are defined with two scalar parameters (*λ*,*β*); *λ* is the scale parameter and *β* is the shape parameter. The hazard function for the Weibull model is increasing if *β *> 1, decreasing if *β *< 1 and constant if *β *= 1 where it reduces to the exponential distribution. The hazard function for the log-logistic model is decreasing if *β *< 1 and has a single maximum if *β *> 1. We therefore consider the families of the exponential, Weibull and log-logistic distributions.

**Table 1:** Exponential, Weibull and log-logistic distributions

**Distribution**	**Exponential**	**Weibull**	**Log-logistic**
Density	*f *(*x*) = *λe*^-*λ* *x*^	$(x)=\lambda \beta {\left(\lambda x\right)}^{\beta -1}e^{\left(-{\left(\lambda x\right)}^{\beta }\right)}$	$f(x)=\frac{\lambda \beta {\left(\lambda x\right)}^{\beta -1}}{{\left(1+{\left(\lambda x\right)}^{\beta }\right)}^{2}}$
Support	*x *> 0	*x *> 0	*x *> 0
Parameter(s)	*λ *> 0	*λ *> 0	*λ *> 0
		*β *> 0	*β *> 0

The times-to-onset were generated from these three distributions. Two values of *λ* were considered for the exponential distribution: 0.05 and 1. The same values were used for the scale parameter *λ* of the Weibull and log-logistic distributions. For the shape parameter *β*, the values 0.5 and 2 were chosen. The truncation times were uniformly distributed in [0,*τ*]. Survival and truncation times were independently generated. For a chosen value of *p*, with *p* representing the probability of *X* falling within the observable values interval [0, *τ*], the parameter *τ* was determined as *P *(*X*< *τ*) = *p*. The probability 1 - *p* is also a lower bound of the actual proportion of truncated data *P *(*X*> *T*), the truncation time *T* being randomly generated. The probability *p* was chosen in {0.25, 0.50, 0.80}. The sample size *n* was chosen in {100, 500}. For each drawn pair (*X*,*T*), if the time-to-onset was shorter than the truncation time, then the pair was included in the data. If not, another pair (*X*,*T*) was generated. Pairs were generated until the sample size of observations included was equal to *n*.

Parametric likelihood maximization with and without considering right truncation were performed for each generated sample. An iterative algorithm is necessary to solve this optimization problem except for the naive exponential estimation. Calculations were made with the R [24] function *maxLik* from the package **maxLik**. For each set of simulation parameters, 1000 replications were run.

### Application study

We analyzed 64 French cases of lymphoma that occurred after anti TNF- *α* treatment using the national pharmacovigilance database at the date of February 1, 2010 [25]. The population included patients suffering from rheumatoid arthritis, Crohn’s disease, ankylosing spondylitis, psoriatic arthritis, psoriasis, Sjögren’s syndrome, dermatomyositis, polymyositis or polyarthropathy and exposed to one or (successively) more of the three anti TNF- *α* available at the study date: etanercept, adalimumab and infliximab. The occurrence of a malignant lymphoma was confirmed by histopathological analysis. Marketing authorization was obtained in August 1999 for infliximab, in September 2002 for etanercept and in September 2003 for adalimumab. These 64 adverse effects occurred between July 2001 and October 2009. None of the survival or truncation times was missing in the database. The observed maximum truncation time was 529 weeks.

All anti TNF-agents taken together, we derived the parametric maximum likelihood estimates and secondarily corresponding estimated mean times, with and without considering right truncation, for the exponential, Weibull and log-logistic distributions. For completeness, we also derived the non-parametric maximum likelihood estimation.

The French pharmacovigilance database is developed by the French drug agency (*Agence Nationale de Sécurité du Médicament et des produits de santé, ANSM*) and is not publicly available. It is build up and used on an ongoing basis by the network of regional pharmacovigilance centres, which have a direct access to the data. This set of data has already been extracted for another study [25] with the authorization of the ANSM and the network of regional centres, according to the internal rule.

## Results

### Simulation study

For each set of simulations parameters, for both approaches and for both parameters, the bias and the mean squared error of the parametric maximum likelihood estimator, based on the 1000 replications, were calculated as well as the proportion of replications where the estimate is larger than the true value. As the iterative algorithm may fail to find a maximum, those three quantities were actually calculated on the replications where there was no problem of maximization. The mean squared error is a measure of the dispersion of the estimator around the true value of the parameter - the smaller the better - and is used for global comparative purposes between two estimation procedures, as it incorporates both the variance of the estimator and its bias. The proportion of replications where the estimate is larger than the true value makes it possible to know if the estimators tend to overestimate or underestimate systematically the true value of the parameter.

#### ***Bias and mean squared error***

For both approaches, for all distributions and for both parameters, the smaller is *p*, the larger are the bias and the mean squared error (Tables 2, 3 and 4). This increase with *p* is smaller for the parameter *β* than for the parameter *λ*. These estimators tend to be positively biased. However, the bias might be almost naught for the TBE. The bias and the mean squared error of the naive estimator are always larger than the bias and the mean squared error of the TBE, but to a lesser extent for the parameter *β*. When the sample size *n* increases, the bias and the mean squared error are almost constant for the naive estimator, while for the TBE, they decrease clearly (Tables 2, 3 and 4). The naive estimator might be unacceptably large whatever the value of *p*, whereas the TBE shows good performances when *p* is equal to 0.8, and often even less according to the distribution.

**Table 2:** Simulation results: estimations of bias and mean squared error for the exponential model

				**Naive estimator**		**TBE**	
***λ***	***p***	***n***	**BIAS(**λ^**)**	**MSE(**λ^**)**	**BIAS(**λ^**)**	**MSE(**λ^**)**	**NPM**
0.05	0.25	100	0.498	0.250	0.030	0.005	224
		500	0.498	0.248	0.007	0.001	79
0.05	0.50	100	0.195	0.038	0.008	0.001	85
		500	0.193	0.037	<0.001	<0.001	1
0.05	0.80	100	0.073	0.005	<0.001	<0.001	2
		500	0.072	0.005	<0.001	<0.001	0
1	0.25	100	10.06	102	0.462	2.17	72
		500	9.95	99	0.046	0.48	10
1	0.50	100	3.91	15.4	0.126	0.49	29
		500	3.86	14.9	-0.022	0.12	0
1	0.80	100	1.45	2.16	0.004	0.11	0
		500	1.45	2.11	0.004	0.02	0

The mean squared error formula is MSE(λ^)=Var(λ^)+(BIAS(λ^))2. Calculations were made on the replications where there was no problem of maximization. In the last column appear the number of problems of maximization for the truncation-based approach. There was no problem of maximization for the naive approach. *Abbreviations*: *TBE* truncation-based estimator, *MSE* mean squared error, *NPM* number of maximization problems.

**Table 3:** Simulation results: estimations of bias and mean squared error for the Weibull model

						**Naive estimator**		**TBE**				
					λ^		β^		λ^		$\hat{\beta }$	
***λ***	***β***	***p***	***n***	**BIAS**	**MSE**	**BIAS**	**MSE**	**BIAS**	**MSE**	**BIAS**	**MSE**	**NPM**
0.05	0.5	0.25	100	4.04	16.7	0.200	0.044	0.465	0.51	0.046	0.007	312
			500	3.95	15.6	0.195	0.039	0.106	0.04	0.013	0.001	201
0.05	0.5	0.50	100	0.762	0.60	0.167	0.031	0.068	0.018	0.024	0.005	172
			500	0.747	0.56	0.164	0.028	0.015	0.003	0.003	0.001	22
0.05	0.5	0.80	100	0.160	0.027	0.119	0.017	0.008	0.002	0.009	0.004	9
			500	0.156	0.025	0.113	0.013	0.001	<0.001	0.001	<0.001	0
1	0.5	0.25	100	80.4	6612	0.201	0.044	8.68	183	0.046	0.007	300
			500	78.9	6249	0.194	0.038	2.07	17	0.012	0.001	186
1	0.5	0.50	100	15.0	233	0.174	0.034	1.53	7.99	0.031	0.006	163
			500	15.0	225	0.164	0.028	0.32	1.17	0.003	0.001	24
1	0.5	0.80	100	3.20	10.8	0.117	0.017	0.16	0.67	0.007	0.004	13
			500	3.15	10.0	0.112	0.013	0.041	0.15	<0.001	<0.001	0
0.05	2	0.25	100	0.121	0.015	0.354	0.16	<0.001	0.002	0.097	0.075	8
			500	0.120	0.014	0.333	0.12	-0.004	0.001	0.020	0.016	2
0.05	2	0.50	100	0.065	0.004	0.278	0.11	-0.004	<0.001	0.047	0.074	6
			500	0.064	0.004	0.264	0.08	-0.002	<0.001	0.004	0.016	0
0.05	2	0.80	100	0.032	0.001	0.182	0.063	<0.001	<0.001	0.046	0.063	1
			500	0.032	0.001	0.157	0.031	<0.001	<0.001	0.008	0.014	0
1	2	0.25	100	2.41	5.84	0.364	0.17	0.090	0.79	0.10	0.075	1
			500	2.41	5.79	0.336	0.12	-0.082	0.38	0.02	0.015	0
1	2	0.50	100	1.29	1.68	0.283	0.12	-0.073	0.33	0.052	0.069	3
			500	1.29	1.65	0.261	0.07	-0.065	0.12	-0.002	0.017	0
1	2	0.80	100	0.638	0.41	0.186	0.065	-0.024	0.086	0.045	0.064	0
			500	0.636	0.40	0.154	0.030	-0.007	0.014	0.004	0.013	0

The mean squared error formula is MSE(λ^)=Var(λ^)+(BIAS(λ^))2. Calculations were made on the replications where there was no problem of maximization. In the last column appear the number of problems of maximization for the truncation-based approach. There was no problem of maximization for the naive approach. *Abbreviations *: *TBE *truncation-based estimator, *MSE *mean squared error, *NPM *number of maximization problems.

**Table 4:** Simulation results: estimations of bias and mean squared error for the log-logistic model

						**Naive estimator**		**TBE**				
					λ^		β^		λ^		β^	
***λ***	***β***	***p***	***n***	**BIAS**	**MSE**	**BIAS**	**MSE**	**BIAS**	**MSE**	**BIAS**	**MSE**	**NPM**
0.05	0.5	0.25	100	6.45	44	0.384	0.16	0.258	0.25	0.041	0.008	217
			500	6.33	40	0.372	0.14	0.043	0.01	0.005	0.001	52
0.05	0.5	0.50	100	1.05	1.2	0.319	0.108	0.045	0.012	0.020	0.006	22
			500	1.02	1.1	0.308	0.096	0.009	0.001	0.003	0.001	0
0.05	0.5	0.80	100	0.165	0.031	0.195	0.041	0.008	0.001	0.008	0.004	0
			500	0.158	0.026	0.189	0.036	0.001	<0.001	0.001	<0.001	0
1	0.5	0.25	100	129	17533	0.383	0.15	5.06	87	0.042	0.008	207
			500	127	16217	0.374	0.14	1.01	6	0.008	0.001	41
1	0.5	0.50	100	21.0	467	0.317	0.106	0.93	5.0	0.019	0.006	43
			500	20.5	426	0.308	0.096	0.20	0.6	0.004	0.001	0
1	0.5	0.80	100	3.31	12	0.201	0.044	0.209	0.55	0.016	0.005	0
			500	3.17	10	0.190	0.037	0.037	0.09	0.002	<0.001	0
0.05	2	0.25	100	0.150	0.022	1.06	1.2	<0.001	0.001	0.08	0.085	4
			500	0.149	0.022	1.04	1.1	-0.001	<0.001	0.01	0.018	0
0.05	2	0.50	100	0.079	0.006	0.932	0.94	<0.001	<0.001	0.06	0.094	5
			500	0.078	0.006	0.903	0.83	<0.001	<0.001	0.01	0.017	0
0.05	2	0.80	100	0.035	0.001	0.665	0.50	<0.001	<0.001	0.03	0.078	0
			500	0.035	0.001	0.649	0.43	<0.001	<0.001	0.01	0.013	0
1	2	0.25	100	2.99	9.0	1.07	1.2	0.024	0.57	0.08	0.089	0
			500	2.98	8.9	1.04	1.1	-0.028	0.20	0.01	0.020	0
1	2	0.50	100	1.57	2.49	0.943	0.96	0.007	0.19	0.063	0.095	1
			500	1.56	2.45	0.896	0.82	-0.013	0.04	0.004	0.018	0
1	2	0.80	100	0.702	0.50	0.668	0.50	0.004	0.042	0.045	0.072	0
			500	0.693	0.48	0.648	0.43	0.004	0.007	0.015	0.013	0

The mean squared error formula is MSE(λ^)=Var(λ^)+(BIAS(λ^))2. Calculations were made on the replications where there was no problem of maximization. In the last column appear the number of problems of maximization for the truncation-based approach. There was no problem of maximization for the naive approach. *Abbreviations *: *TBE *truncation-based estimator, *MSE *mean squared error, *NPM *number of maximization problems.

#### ***Proportion of replications where the estimator is larger than the true value***

For both approaches, for all distributions and for both parameters, Tables 5, 6 and 7 show that the naive estimator of *λ* appears to be almost always larger than the theoretical value *λ*, and that this is not far from being true for the naive estimator of *β*. This suggests that the naive estimator of *λ* might be almost surely larger than the true value of the parameter, which would be a - non desirable - statistical feature of the naive estimator.

**Table 5:** Simulation results: proportion of replications where the maximum likelihood estimator is larger than the true value of the parameter for the exponential model

***λ***	***p***	***n***	**Naive estimator**	**TBE**
0.05	0.25	100	100%	61.6%
		500	100%	55.3%
0.05	0.50	100	100%	55.3%
		500	100%	50.4%
0.05	0.80	100	100%	51.1%
		500	100%	51.7%
1	0.25	100	100%	54.8%
		500	100%	50.7%
1	0.50	100	100%	53.2%
		500	100%	48.0%
1	0.80	100	100%	50.0%
		500	100%	51.0%

Calculations were made on the replications where there was no problem of maximization. *Abbreviations *: *TBE *truncation-based estimator.

**Table 6:** Simulation results: proportion of replications where the maximum likelihood estimator is larger than the true value of the parameter for the Weibull model

				**Naive estimator**		**TBE**	
***λ***	***β***	***p***	***n***	λ^>λ	β^>β	λ^>λ	β^>β
0.05	0.5	0.25	100	100%	100%	81.4%	71.9%
			500	100%	100%	64.6%	64.5%
0.05	0.5	0.50	100	100%	100%	63.3%	60.1%
			500	100%	100%	53.4%	51.0%
0.05	0.5	0.80	100	100%	99.6%	52.0%	53.3%
			500	100%	100%	48.6%	51.6%
1	0.5	0.25	100	100%	100%	79.3%	76.0%
			500	100%	100%	62.0%	61.2%
1	0.5	0.50	100	100%	100%	65.9%	64.6%
			500	100%	100%	53.8%	51.8%
1	0.5	0.80	100	100%	99.5%	52.7%	52.2%
			500	100%	100%	51.9%	50.6%
0.05	2	0.25	100	100%	98.1%	52.1%	61.6%
			500	100%	100%	52.2%	53.7%
0.05	2	0.50	100	100%	94.2%	51.6%	53.3%
			500	100%	100%	50.6%	51.0%
0.05	2	0.80	100	100%	85.4%	56.1%	55.8%
			500	100%	97.9%	52.2%	49.6%
1	2	0.25	100	100%	98.2%	56.2%	62.5%
			500	100%	99.9%	50.1%	54.8%
1	2	0.50	100	100%	94.3%	53.9%	54.2%
			500	100%	99.9%	47.1%	48.1%
1	2	0.80	100	100%	85.3%	54.1%	54.2%
			500	100%	97.9%	52.7%	52.2%

Calculations were made on the replications where there was no problem of maximization. *Abbreviations *: *TBE *truncation-based estimator.

**Table 7:** Simulation results: proportion of replications where the maximum likelihood estimator is larger than the true value of the parameter for the log-logistic model

				1**Naive estimator**		**TBE**	
***λ***	***β***	***p***	***n***	λ^>λ	β^>β	λ^>λ	β^>β
0.05	0.5	0.25	100	100%	100%	67.2%	67.7%
			500	100%	100%	53.6%	52.0%
0.05	0.5	0.50	100	100%	100%	55.4%	57.5%
			500	100%	100%	51.1%	52.0%
0.05	0.5	0.80	100	100%	100%	51.1%	53.2%
			500	100%	100%	50.8%	51.5%
1	0.5	0.25	100	100%	100%	67.7%	66.1%
			500	100%	100%	55.9%	56.1%
1	0.5	0.50	100	100%	100%	54.9%	57.2%
			500	100%	100%	53.4%	53.4%
1	0.5	0.80	100	100%	100%	55.1%	56.5%
			500	100%	100%	51.9%	52.0%
0.05	2	0.25	100	100%	100%	53.2%	55.9%
			500	100%	100%	51.8%	51.8%
0.05	2	0.50	100	100%	100%	55.0%	54.2%
			500	100%	100%	53.3%	52.2%
0.05	2	0.80	100	100%	100%	50.3%	51.5%
			500	100%	100%	53.9%	54.4%
1	2	0.25	100	100%	100%	52.7%	56.1%
			500	100%	100%	53.3%	51.0%
1	2	0.50	100	100%	100%	54.3%	56.4%
			500	100%	100%	50.1%	49.5%
1	2	0.80	100	100%	100%	52.0%	53.7%
			500	100%	100%	52.9%	55.0%

Calculations were made on the replications where there was no problem of maximization. *Abbreviations *: *TBE *truncation-based estimator.

### Application study

Table 8 presents the estimates of the parameters for the three models and both approaches. There was no problem of maximization. The naive estimates are always larger than the truncation-based estimates. From the simulation results, it might be thought that the naive estimator overestimates the true values of parameters *λ* and *β*, and that the size of the bias is related to the unknown probability *p*. Estimations of the parameters for the truncation-based approach make it possible to estimate *p *by calculating F(t∗=529;θ^TBE). However, estimates of *p *are different according to the model (Table 8). In particular, for the Weibull model, the estimate is large (p^ = 0.98). The larger is p^, the closer are the naive and the truncation-based estimates.

**Table 8:** **Parameter estimation and estimated mean time-to-onset for 64 cases of lymphoma that occurred after anti TNF- *α *** **treatment**

	**Naive estimator**			**TBE**				
**Distribution**	λ^	β^	**Expectation (weeks)**	λ^	β^	p^	**Expectation (weeks)**	
Exponential	0.00739	-	135	0.00172	-	0.60	581	[264,7528]^*^
Weibull	0.00666	1.55	135	0.00468	1.49	0.98	193	[150,432]^*^
Log-logistic	0.00890	2.06	171	0.00408	1.53	0.76	567	[207,1.8 ×10^12^]^*^

^*^95% confidence intervals calculated using BCa simple bootstrap method based on 5000 replicates.

$$\hat{p}=F(t^{*}=529;({\hat{\lambda }}_{\text{TBE}},{\hat{\beta }}_{\text{TBE}}))$$ .

*Abbreviations *: *TBE *truncation-based estimator.

**Figure 2:**
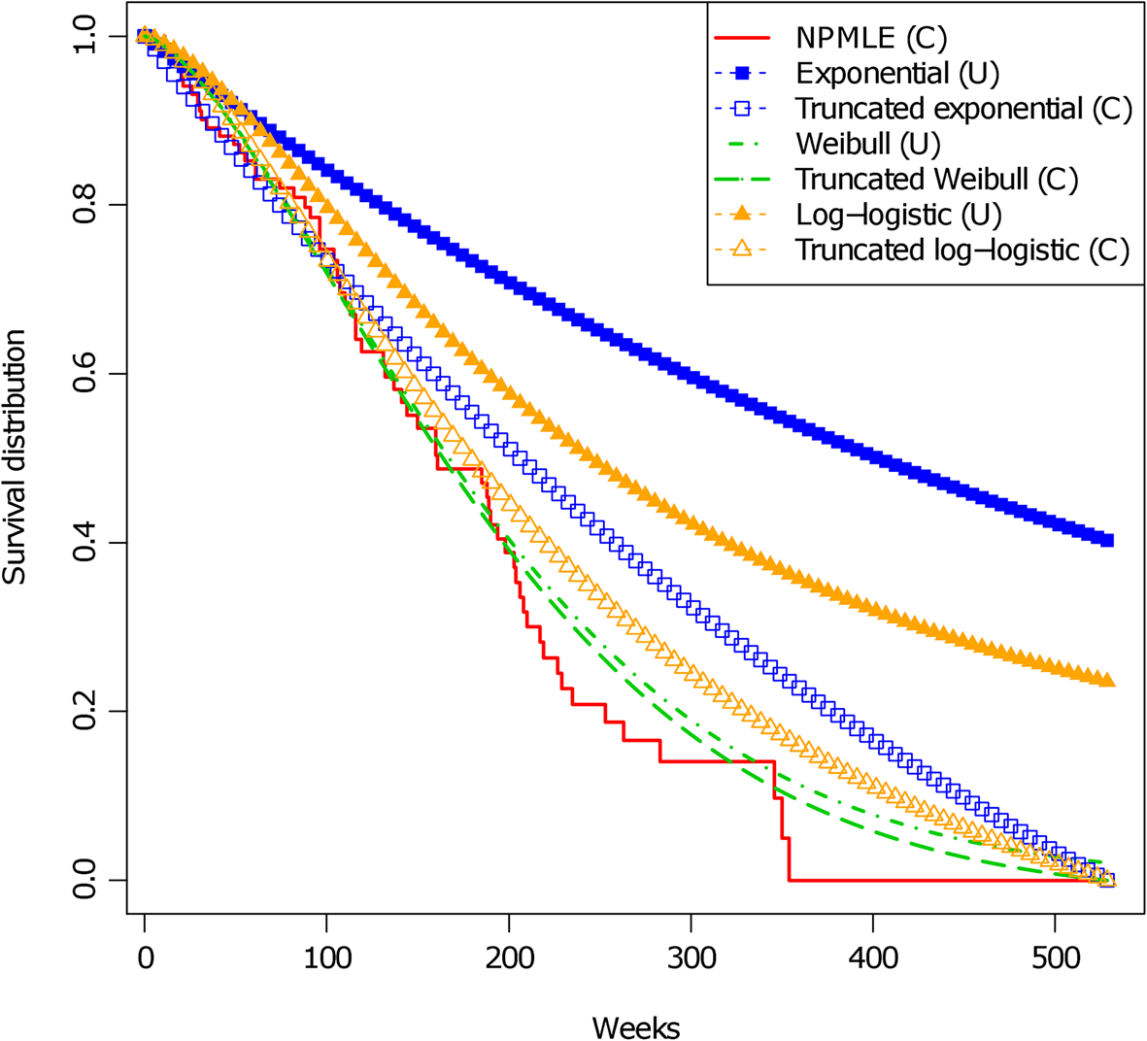
**Right truncation-based estimations of time-to-onset of lymphoma that occurred after anti TNF-*α *treatment.** Data include 64 cases. Three models are fitted: exponential, Weibull and log-logistic. Estimations of the conditional survival function (C), estimations of the unconditional survival function (U) and the non-parametric maximum likelihood estimation of the survival function (NPMLE) are displayed.

Figure 2 shows the non-parametric maximum likelihood estimation of the conditional survival function, $\hat{\frac{F(x)}{F(529)}}$, and the parametric maximum likelihood estimation of the conditional, F(x;θ^TBE)F(529;θ^TBE), and unconditional, F(x;θ^TBE), survival functions for the truncation-based approach for these data. The estimations of the conditional survival functions are always closer to the non-parametric estimation than the estimations of the unconditional survival functions. The conditional and unconditional estimations of the Weibull survival functions are almost similar because the estimate of *p* is about 1. This figure shows that the estimation of the conditional Weibull survival function is closer to the non-parametric maximum likelihood estimation of the conditional survival function than the estimations of the conditional exponential and conditional log-logistic survival functions. Thus, Weibull could be a reasonable candidate model to describe the data.

**Figure 3:**
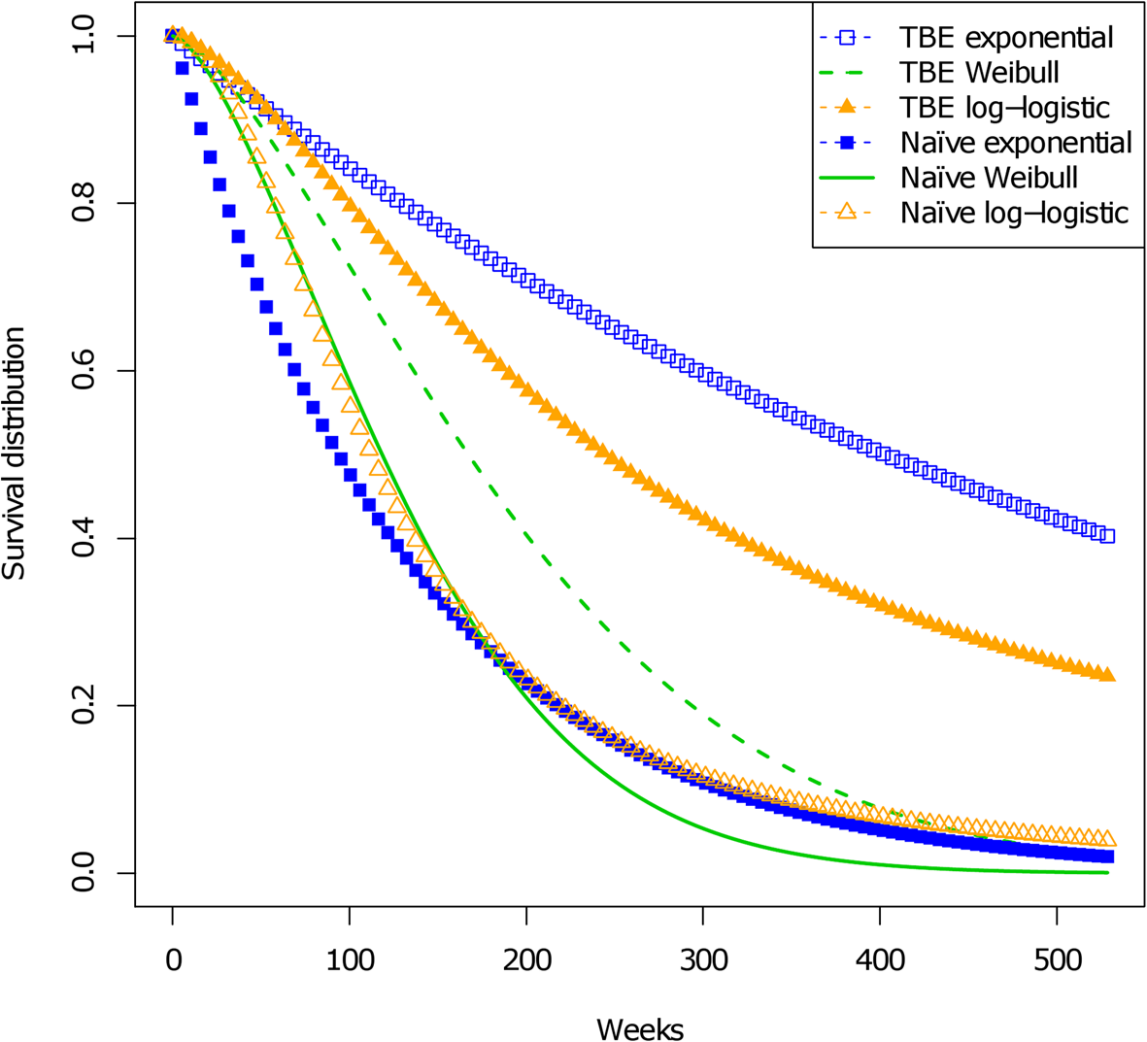
**Naive and right truncation-based estimations of time-to-onset of lymphoma that occurred after anti TNF-*α *treatment.** Data include 64 cases. Three models are fitted: exponential, Weibull and log-logistic. Estimations of the unconditional survival function for the naive approach (Naive) and for the truncation-based approach (TBE) are displayed.

Figure 3 shows the parametric maximum likelihood estimation of the unconditional survival function for both approaches. The distance between both survivals, naive and truncation-based, decreases with the estimated probability p^ (in the order: exponential, log-logistic and Weibull). Furthermore, the survival functions from the truncation-based estimates are always above the survival functions from the naive estimates, which is consistent with the naive estimator overestimating the true values of the parameters *λ *and *β*. Even for the Weibull model, *i.e. *the model with the largest p^, the estimated expected time-to-onset would be 135 weeks with the naive approach and 193 weeks with the truncation-based estimates, which corresponds to a markedly large gap (Table 8). For completeness, we also calculated the 95% simple bootstrap confidence intervals of the expected time (BCa method) [26,27] based on 5000 bootstrap samples, for the truncation-based approach. They do not include the naive estimated mean time, whatever the fitted model, and even though these confidence intervals are extremely wide.

## Discussion and conclusions

In drug safety assessment, the temporal relationship between drug administration and time-to-onset is of utmost relevance. A better understanding of the underlying mechanism of the occurrence of an adverse effect is crucial, as it could allow the identification of particular groups of patients at risk and of particular risk time-windows in the course of a treatment and lead to preventing or diagnosing earlier the occurrence of adverse reactions. In this framework, the time-to-onset of an adverse drug reaction constitutes an essential feature to be analyzed. Its accurate estimation and modeling could help in understanding the mechanism of a drug’s action.

As rare adverse effects are not generally identified by cohort studies of exposed patients but from spontaneous reporting systems, we investigated with a simulation study the accuracy of estimates that can be obtained from these data in a parametric framework. As one can only estimate a conditional distribution function in a non-parametric setting, the non-parametric maximum likelihood estimator is of rather little interest for pharmacovigilance people. For a finite sample size, the simulations show that, whatever the approach, naive or truncation-based, the parametric maximum likelihood estimator may be positively biased and that this bias and the corresponding mean squared error increase when the theoretical probability *p* for the time-to-onset to fall within the observable values interval decreases. However, for a fixed value of *p*, the bias and the mean squared error are always larger when the right truncation is not considered than when it is, and the gap may be large. In addition, bias and mean squared error might in some instances (Weibull, log-logistic) be unacceptably large for the naive approach, even for a large value of *p*, while with a probability *p* of 0.8, or sometime even less, the TBE shows good performances. Asymptotically, the naive estimator may not be unbiased because the bias and the mean squared error seem to be constant with the sample size and the maximization is based on a misleading likelihood, while the bias and the mean squared error for the TBE decrease as the sample size increases. Therefore, even if the sample size is large, the gap between both estimators does not disappear and the truncation-based approach should be used.

The probability *p* plays an important role in the estimation of the distribution of the time-to-onset of adverse reaction for right-truncated data. Knowledge exists on a range of possible pharmacological mechanisms. It is thus possible to get a rough idea of the fraction of potentially missed cases (the adverse reactions of treated patients that have yet to occur) and then to decide on the relevance of the time of analysis. Spontaneous reports result from three processes: the occurrence case process, its diagnosis and the reporting process. It is well known that under-reporting is widespread, even for serious events. In addition, factors of under-reporting include the seriousness of the effect, the age of the patient and the novelty of the effect, but also time-related variables such as the length of marketing or the time since exposure [28-33]. In the approach proposed here, it is assumed that the under-reporting is uniform. Such a hypothesis might not always be acceptable. However, with long-term effects such as lymphoma and a homogeneous observation period within the marketing life of the product, non-stationarity of reporting is unlikely.

Problems of maximization may arise when right truncation is taken into account. The smaller is *p*, the more the iterative algorithm is likely to fail. Some papers mentioned the existence of a problem in the parametric likelihood maximization and explained that, because of right truncation, the likelihood may be flat and the maximum may be difficult to find [21,34-36].

For the 64 cases of lymphoma after anti TNF- *α* treatment, there was no problem of convergence of the iterative algorithm. Both estimates, naive and truncation-based, were available for each fitted model. From the truncation-based estimates, it is possible to estimate *p*. Here it ranges from 0.98 (Weibull) to 0.60 (exponential). Since this probability is unknown, the non-parametric maximum likelihood estimation estimates only the distribution function conditional on the time-to-event being less than the maximum observed truncation time. However, although conditional, the non-parametric estimate is a reference that provides an idea of how the data fit a given model. We followed the graphical procedure for checking goodness-of-fit for right-truncated data suggested by Lawless (2003) that is based on the non-parametric maximum likelihood estimator and consists in plotting the conditional fitted parametric survivals together with the non-parametric estimation [36]. Here, the conditional Weibull survival function seems the closest to the non-parametric estimation. This finding underlines the interest for developing goodness-of-fit tests adapted to right-truncated data. While only three families of distributions were considered for the present simulation study, other families could be explored such as the gamma or the log-normal families or mixture models. For instance, in more complex situations, the treatment might be a combination of drugs, each of them inducing the effect but in a different time window. In that case, the hazard function may vary several times and a family of more complex distributions could be of greater interest. Additionally, we chose to consider the truncation times as deterministic, which is equivalent to working on conditional distributions for the likelihood. However, another possible approach is to consider the truncation time as a random variable and to study the random pair (*X*,*T*) where *X* is the survival time and *T* is the truncation time [37-39].

Finally, improvement of time-to-onset distribution assessment could make it possible to compare two drug profiles or more generally to assess risk factors with regression models.

## Competing interests

The authors declare that they have no competing interests.

## Authors’ contributions

FL, JYD and PTB conceived and designed the work. FL implemented the simulations, performed data analysis and wrote the initial draft of the manuscript. HT and FH made the extraction of the data from the national pharmacovigilance database. All authors contributed to the interpretation of the results of the data analysis. All authors reviewed and revised the draft version of the manuscript. All authors read and approved the final version of the manuscript.

## Pre-publication history

The pre-publication history for this paper can be accessed here:

http://www.biomedcentral.com/1471-2288/14/17/prepub
